# Increased serum exosomal miR-134 expression in the acute ischemic stroke patients

**DOI:** 10.1186/s12883-018-1196-z

**Published:** 2018-12-04

**Authors:** Jingxia Zhou, Lin Chen, Bocan Chen, Shaozhu Huang, Chaosheng Zeng, Hairong Wu, Cong Chen, Faqing Long

**Affiliations:** Department of Neurology, The Second Affiliated Hospital of Hainan Medical University, Haikou, 570311 Hainan Province People’s Republic of China

**Keywords:** Acute ischemic stroke, Exosome, miR-134, National Institutes of Health stroke scale scores, Infarct volume

## Abstract

**Background:**

The exosomal miRNAs have been emerged as biomarkers and therapeutic targets for various diseases, however, the function of exosomal miRNAs in stroke remains largely unknown.

**Methods:**

The blood samples from acute ischemic stroke (AIS) patients and normal controls were collected. The exosomes were isolated from the blood samples, which were confirmed by electron microscopy and western blot with the specific exosomes biomarker CD9, CD63 and Tsg101.

**Results:**

RT-qPCR analysis showed that exosomal miR-134 was significantly increased in AIS patients within 24 h after stroke onset compared with that of control group. Highly expressed exosomal miR-134 was correlated with the National Institutes of Health Stroke Scale (NIHSS) scores, infarct volume and positively associated with the worse prognosis of the stroke patients. Additionally, the exosomal miR-134 was strong positively correlated with the expression of serum interleukin 6 (IL-6) and plasma high-sensitivity C relative protein (hs-CRP). The receiver operating characteristic (ROC) curve suggested that miR-134 might be a potential factor to discriminate AIS patients from non-stroke controls.

**Conclusions:**

The exosomal miR-134 as a possible novel biomarker for the diagnosis and prognosis of stroke.

## Background

Stroke is the main cause of neurological dysfunction, which has been considered as the second most common cause of death worldwide and brings heavy burden to the society and family [1–6]. Although remarkable progress has been made in the treatment of stroke, the diagnosis of stroke is still difficult as determining the cause of stroke remains complicated in up to 25% of the patients. Currently, there are no blood-based biomarkers with clinical utility for acute ischemic stroke. Therefore, exploring novel biomarkers involved in the pathogenesis of stroke still remains a challenge.

Tissue responding to the injury caused by stroke and the neuron remodeling after ischemia have been considered as a promising strategy for stroke treatment [7–10]. This process requires the alternation of gene expression in neurons. MicroRNAs (miRNAs) are characterized as non-coding RNAs that inhibit the gene expression via post- transcriptionally regulating the targets via binding the 3′-untranslational region [11–14]. MiRNAs are key regulators of the cell proliferation, differentiation and migration [15]. Aberrant expression of miRNAs has been found in a variety of human diseases. Recent studies showed that circulating miRNAs are highly stable and detectable in the serum of patients with ischemic stroke [16–19]. Up-regulated or down-regulated circulating miRNAs has been identified in stroke patients, which suggested that circulating miRNAs might be a potential biomarkers and therapy targets in stroke.

It has been reported that miRNA can be secreted from cells into the body fluids via exosomes [20–23]. Exosomes are identified as 30–100 nm vesicles that are released into the extracellular space, which deliver protein, mRNA, miRNA or lipid from parent cells to recipient cells [24]. Exosomal miRNAs establish the communication between cell-to-cell and are involved in regulating different biological processes via modulating the expression of target genes. Notably, the critical role of exosomal miRNAs in the development of stroke is emerging [17, 19, 25]. Recent study showed that the plasma exosomal miR-422a and miR-125b-2-3p were potential blood-base biomarkers for monitoring and diagnosing of IS patients [26]. Additionally, the exosome mediated delivery of miR-124 promoted the cortical neurogenesis after stroke [27]. On the other hand, aberrant release of exosomal miRNAs leads to the alternation of gene expression that may induce the initiation and development of cerebral ischemia reperfusion injury. Among these miRNAs, miR-134 was a brain-specific miRNA that was associated with the development of dendritic and synaptic spine [28]. It has been reported that miR-134 was differentially expressed in brain tissues with ischemic injury [29, 30]. In rat model, miR-134 was down-regulated by electroacupuncture and promoted the expression of LIM domain kinase to enhance the synaptic-dendritic plasticity after ischemic stroke [31]. It was also found that overexpression of miR-134 significantly promoted neuron death induced by oxygen-glucose deprivation/reoxygenation by in vitro study [29]. These reports suggested the potential critical roles of miR-134 in regulating cerebral ischemic injury, however, the expression and correlation between miR-134 with the clinical parameters of acute ischemic stroke patients remains largely unknown.

Here, in this study, we detected the expression level of miR-134 in the serum exosomes from the patients with stroke and non-stroke subjects. To determine the clinical significance of miR-134 in stroke, the correlation between the expression of exosomal miR-134 with the National Institutes of Health Stroke Scale (NIHSS) scores, infarct volume, and prognosis of stroke patients were analyzed. The potential application of exosomal miR-134 as a novel biomarker for the diagnosis and therapy of stroke was also discussed.

## Methods

### Clinical sample collection

The 50 stroke patients were recruited between April 2015 and December 2016 in The Second Affiliated Hospital of Hainan Medical University. The ischemic stroke of these patients was confirmed by magnetic resonance imaging (MRI). The severity of stroke was evaluated with the NIHSS score by experienced neurologists. These AIS patients were divided into different Trial of Org 10,172 in Acute Stroke Treatment (TOAST) subtypes including cardioembolism, large artery atherosclerotic stroke and small artery stroke (Table 1). Control group were enrolled from those who underwent an annual medical examination and denied history of stroke attack at The Second Affiliated Hospital of Hainan Medical University. The cell-free serum samples were collected from the peripheral blood via centrifugation and stored at − 80 °C. This study was approved by The Second Affiliated Hospital of Hainan Medical University. Written consent was obtained from each participant. The clinical characteristics of the stroke patients and controls were described in Table 2.

**Table 1:** Stroke patients with different TOAST subtypes

Subtypes	Number
Cardioembolism	23
Large artery atherosclerotic stoke	10
Small artery stroke	17
Total	50

**Table 2:** Baseline clinical characteristics of the AIS patients and normal controls

Parameter	AIS patients (n = 50)	Control (n = 50)	*P* value
Mean age	65.42 ± 10.25	63.33 ± 14.32	> 0.05
Gender (Male/Female)	26/24	22/28	> 0.05
Race (Asian, %)	100%	100%	> 0.05
Ethnicity (Han, %)	100%	100%	> 0.05
Stroke risk factors			
Hypertension	30 (60.0%)	27 (54.0%)	> 0.05
Diabetes mellitus	24 (48.0%)	8 (16.0%)	< 0.01
Atrial Fibrillation	15 (30.0%)	12 (24.0%)	> 0.05
Hyperlipidemia	30 (60.0%)	13 (26.0%)	< 0.01
NIHSS score	7.82 ± 1.36	NA	NA
mRS ≤2	32 (64.0%)	NA	NA
> 2	18 (36.0%)	NA	NA

### Serum collection and exosomes isolation

Five milliliters of fresh whole blood from stroke patients within 24 h of stroke onset or control groups was collected into the promoting coagulating tubes and incubated at 37 °C for 20 min. And then the blood was centrifuged at 1600 *g* for 10 min. The serum was transferred into a new tube and centrifuged at 20,000 g at 4 °C for 15 min. The supernatant was collected and kept at − 80 °C until use. The serum exosomes were extracted with the ExoQuick exosome precipitation solution (System Biosciences, CA, USA). Briefly, the serum sample was centrifuged at 3000 g for 15 min at 4 °C. The supernatant was transferred into a fresh tube and one-fourth volume of ExoQuick Solution was added. The samples were mixed well and incubated at 4 °C for 2 h. And then the mixture was centrifuged at 1500 g for 30 min. The supernatant was discarded and the pellet was re-centrifuged for another 5 min at 4 °C. The exosome-containing pellet was resuspended with nuclease-free water. The size and morphology of exosomes were detected by transmission electron microscopy (TEM, Phillip CM120, 60 kV).

### RT-qPCR analysis

Total RNA extraction from the serum exosomes was performed with the Exosome RNA Purification Kit (System Biosciences Inc., Mountain View, CA, USA) according to the manufacturer’s instructions. The concentration and quality of the RNA was determined using the NanoDrop ND-2000 spectrophotometer (Thermo Scientific Rockford, IL, USA). Single strand cDNA was synthesized with the First Strand cDNA Synthesis Kit (Ferments, Vilnius, Lithuania). The expression of miR-134 was detected by real time PCR using the SYBR Green Master Mix (Applied Biosystems) on the Applied Biosystems 7900 real-time PCR machine. All the reactions were performed in triplicate. The PCR condition was set as the follows: 95 °C for 10 min, and then 42 cycles of 95 °C for 10s and 60 °C for 1 min. The relative expression abundance of miR-134 was calculated with the 2-^∆∆Ct^ method.

### Western blot

Exosomal protein was extracted with the RIPA lysis buffer (Thermo Fisher Scientific, IL, USA). The protein concentration was determined by the Pierce BCA protein assay kit (Thermo Fisher Scientific, IL, USA). 20 μg of protein was loaded and separated by the 12% SDS-PAGE followed by transferred to polyvinylidene fluoride membranes. Afterwards, the membrane was blocked with 5% non-fat milk for 1 h at room temperature (RT) and then incubated with the indicated primary antibodies for 2 h at RT. The bands were detected by incubating the membrane with horse radish peroxidase (HRP)-conjugated secondary antibody for 1 h at RT and visualized using the ECL detection kit (Thermo Fisher Scientific, IL, USA).

### Antibody characterization

The antibodies against CD9 (1:1000, sc-13,118, Santa Cruz Biotechnology), CD63 (1:1000, sc-365,604, Santa Cruz Biotechnology), β-actin (1:2000, sc-47,778, Santa Cruz Biotechnology) and Tsg101 (1:2000, ab30875, Abcam) were purchased from the company.

### Statistical analysis

The statistical analysis was performed with the SPSS software (version 13.0, Chicago, IL, USA). Difference between two groups was compared by the Student’s *t* test and multiple comparisons between more than two groups were determined by the one-way analysis of variance (ANOVA) followed by the Bonferroni test. The Chi-squared test was used to assess the correlation between the expression of miR-134 and clinicopathological factors. The exosomal miR-134 expression with the conventional risk factors of stroke was analyzed by the multivariable logistic regression. The correlation between the expression level of miR-134 and the NIHSS score of the stroke patients was determined with the Pearson’s correlation test. *P* < 0.05 was considered as statistically significant.

## Results

### Identification of the serum exosomes isolated from the AIS patients

**Fig. 1:**
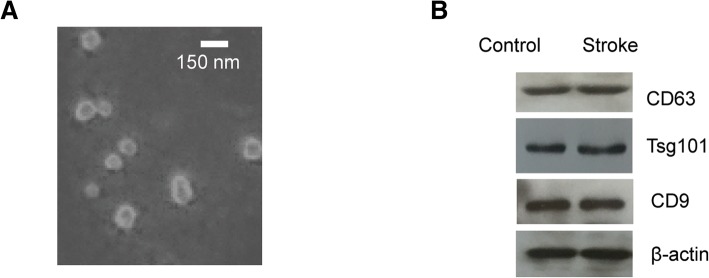
The isolation of exosomes from the serum of AIS patients and controls. **a** The transmission electron microscope of the exosomes purified from the serum. Scale bar, 150 nm. **b** Western blot analysis of the exosomal markers with the indicated antibodies

To isolate exosomes from the blood, ExoQuick kit was used to precipitate the microvesicles. Electron microscopy and western blot analysis have been the gold standard for exosome confirmation. The vesicles isolated in the study displayed a spherical structure with 30–100 nm nano-size (Fig. 1a), which is consistent with the diameter of exosomes. To further confirm this, the expression of CD9, CD63 and Tsg101, the commonly used exosomal markers, was detected by western blot. As shown in Fig. 1b, the protein bands of CD9, CD63, Tsg101 and β-actin were all observed with the indicated antibodies respectively. These data suggested that the isolation method we used was suitable for exosome purification.

### The level of exosomal miR-134 was significantly elevated in stroke patients

**Fig. 2:**
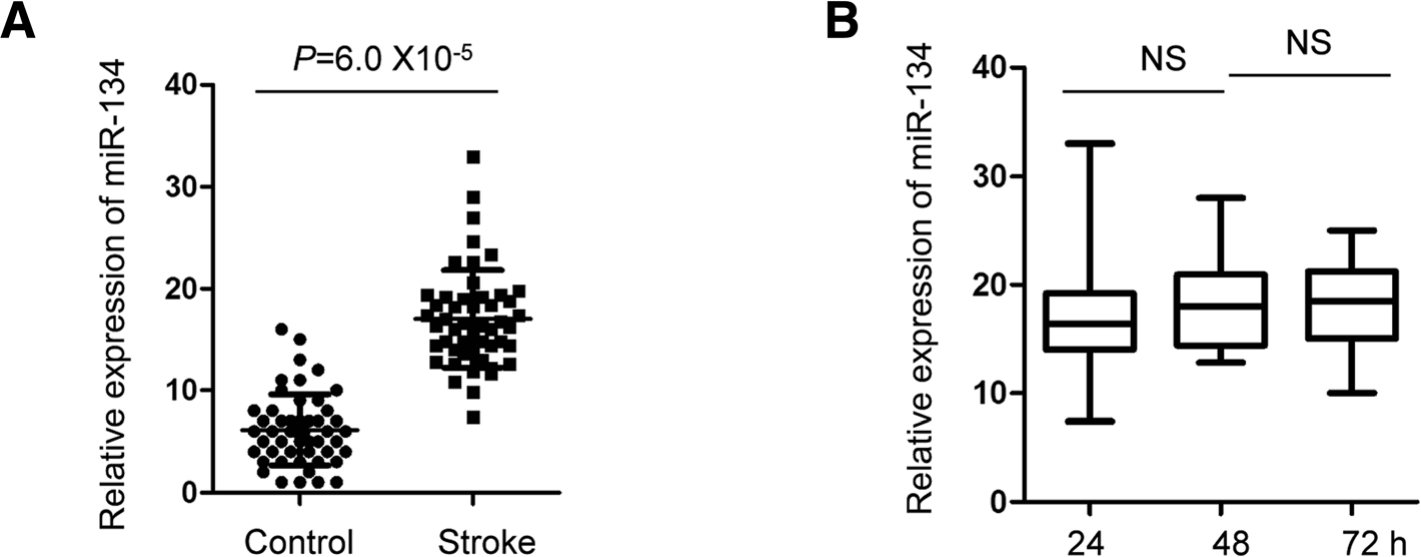
The level of exosomal miR-134 was significantly elevated in stroke patients. **a** RT-qPCR analysis was performed to detect the expression of serum exosomal miR-134 in both the AIS patients and healthy controls. Student’s *t* test, *n* = 50 in stroke group and n = 50 in control group. **b** The expression level of serum exosomal miR-134 of the AIS patients at different time points after the stroke occurred. *N* = 50. Ns, no significance. One-way ANOVA

To compare the expression abundance of exosomal miR-134 in stroke patients and normal controls, RT-qPCR analysis was performed with exosomes isolated from patients and the control group. As shown in Fig. 2a, compared with the control group, the expression of exosomal miR-134 was significantly increased in stroke patients within 24 h of the stroke onset. To detect whether the increased expression of miR-134 was a dynamic process after stroke, the expression level of miR-134 in the circulating exosome was examined at 24-, 48- and 72 h after stroke occurred. The data showed that there was no significant difference for the expression of exosomal miR-134 between different time points of the stroke (Fig. 2b).

### Exosomal miR-134 was a possible biomarker for the diagnosis of AIS

**Fig. 3:**
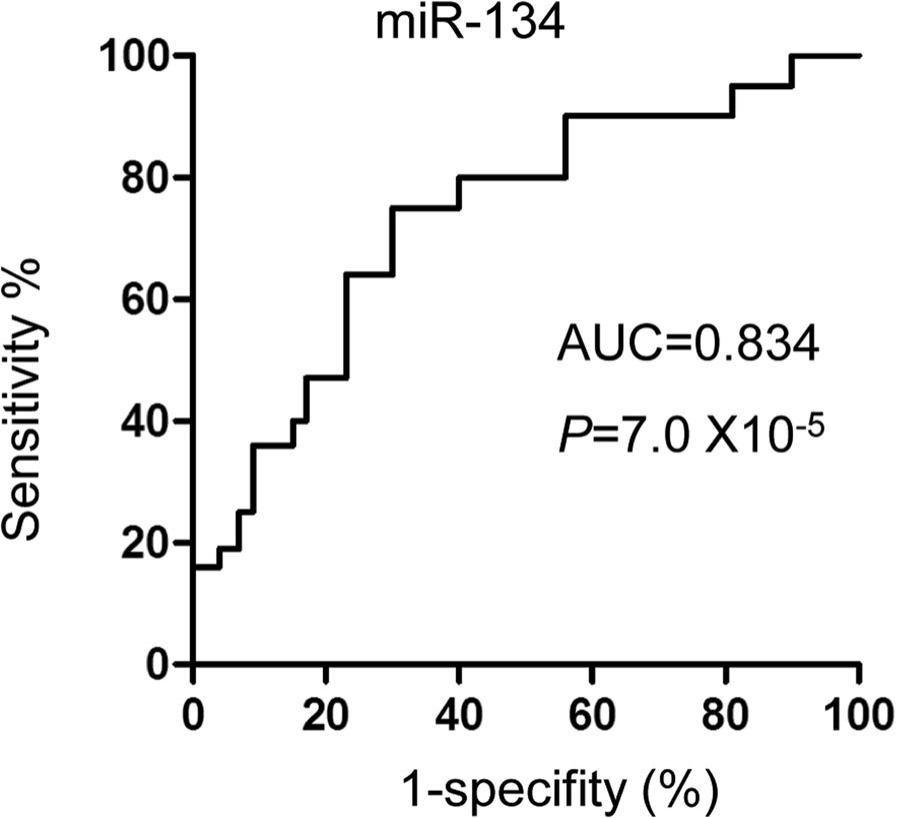
Diagnostic value of serum exosomal miR-134 in AIS. ROC curve was generated based on the level of serum exosomal miR-134 to distinguish AIS patients (n = 50) from normal controls (n = 50). The area under the cure was 0.834 (95% confidence interval, 0.88–0.97)

The high expression of exosomal miR-134 in stroke patients led us to determine whether exosomal miR-134 was an independent risk factor in AIS. To this end, logistic regression analysis was performed to evaluate the impact of diabetes mellitus and hyperlipidemia on the expression of exosomal miR-134 between the stroke patients and normal controls. The data suggested that exosomal miR-134 has the possibility to be an independent risk factor of AIS (Table 3). To determine whether the serum exosomal miR-134 can be considered as a biomarker for the diagnosis of AIS, the receiver operating characteristic (ROC) curve was generated with the expression level of miR-134 in the serum exosomes. As shown in Fig. 3, the area under the curve (AUC) of miR-134 was 0.834 (95% confidence interval, 0.88–0.97), with a sensitivity of 75.3%, a specificity of 72.8%, a positive of 94.0%, and a negative predictive value of 88.0%. This data suggested that miR-134 is a possible potential biomarker to discriminate AIS patients from non-stroke controls.

**Table 3:** Expression of serum exosomal miR-134 and the risk of stroke

Characteristics	*P* value	OR	95% CI
miR-134	0.003	2.668	1.183–6.441
Diabetes mellitus	0.001	2.842	1.514–5.333
Hyperlipidemia	0.047	1.617	1.128–3.038

### The relationship between the expression of exosomal miR-134 and the NIHSS score, infarct volume of the stroke patients

**Fig. 4:**
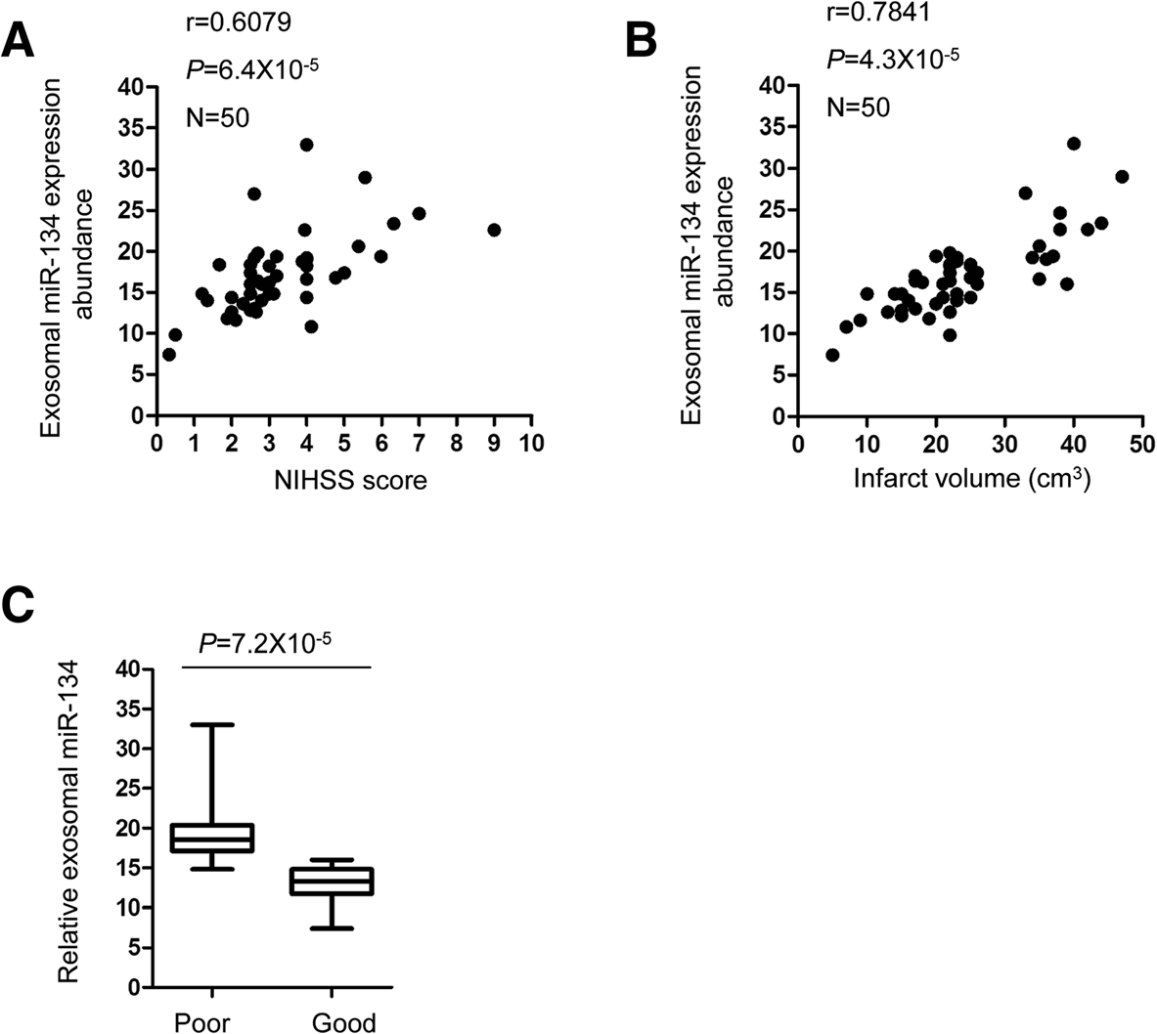
The correlation between the abundance of serum exosomal miR-134 with the NIHSS, infraction volume of AIS patients. **a** The correlation between exosomal level of miR-134 with the NIHSS score of the stroke patients was analyzed by the Person correlation test. N = 50. **b** The correlation between exosomal level of miR-134 with the infarction volume of the stroke patients was analyzed by the Person correlation test. N = 50. **c** The correlation between serum exosomal miR-134 with the outcome of AIS patients. The expression level of miR-134 in AIS patients with good prognosis (modified Rankin Scale (mRS) ≤ 2, *n* = 32) and poor prognosis ((mRS > 2, *n* = 18) was analyzed by the Student’s *t* test

To determine the clinical significance of exosomal miR-134 in the diagnosis of acute ischemic stroke, the correlation between the expression of miR-134 and NIHSS scores, infarct volume was examined by the Pearson correlation test. As presented in Fig. 4a, significant correlation between the level of miR-134 and the NIHSS score was observed. Consistent with this data, the expression abundance of exosomal miR-134 was also significantly correlated with the infarct volume of the acute ischemic stroke patients (Fig. 4b). Additionally, to detect the relationship between the expression of circulating exosomal miR-134 with the 3-month prognosis of the stroke patients, the expression level of miR-134 in patients with good or poor outcome was compared. As shown in Fig. 4c, higher abundance of the exosomal miR-134 was found in stroke patients with poor prognosis. These findings suggested the potential clinical application of exosomal miR-134 in stroke.

### The level of serum exosomal miR-134 was positively correlated with the serum level of IL-6 and plasma hs-CRP

**Fig. 5:**
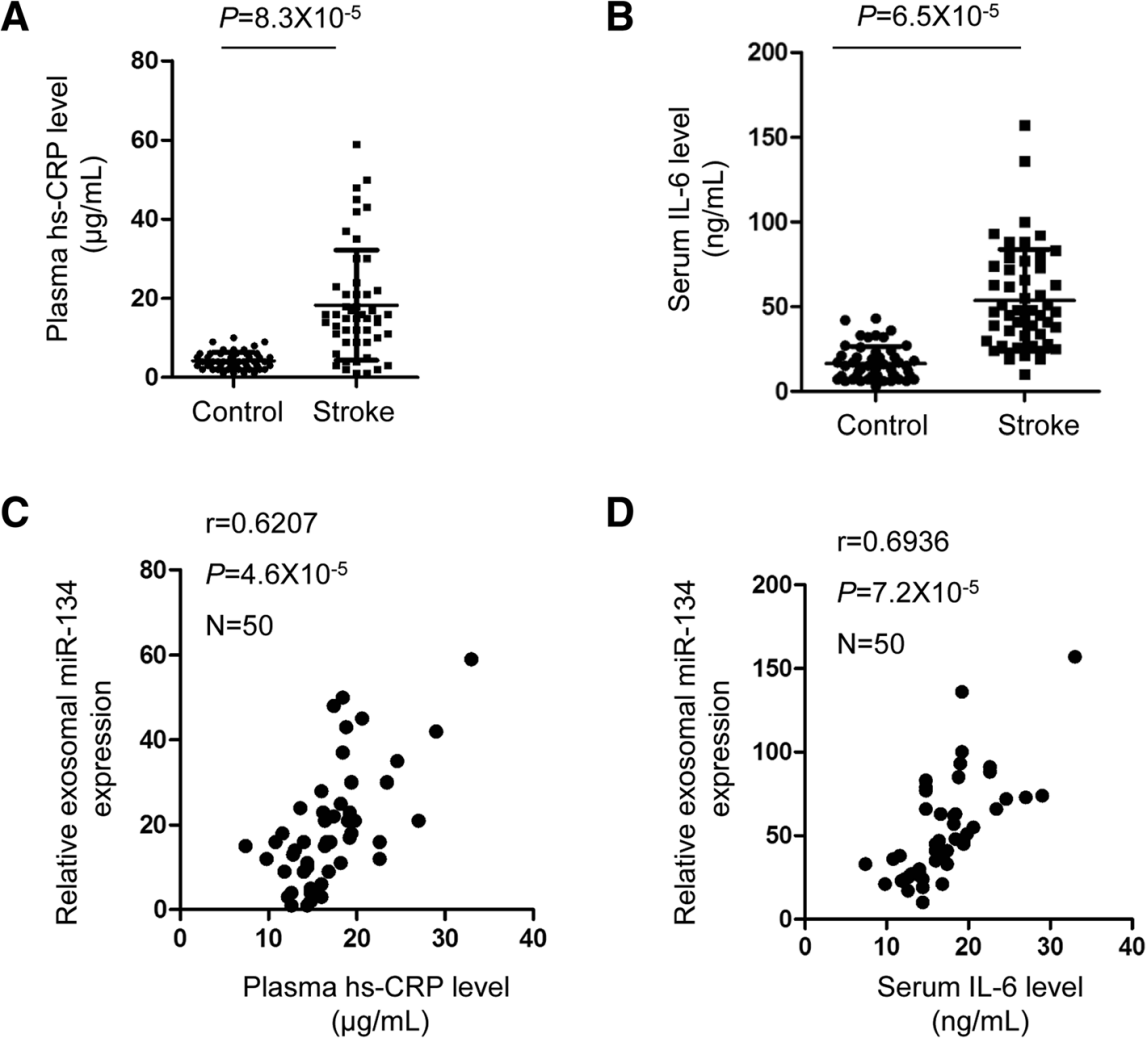
The level of serum exosomal miR-134 was positively correlated with the serum level of IL-6 and plasma hs-CRP. **a**, **b** The levels of plasma hs-CRP and serum IL-6 were detected in AIS patients and control participants. Student’s *t* test. **c**, **d** The exosomal miR-134 was positively correlated with the concentration of plasma hs-CRP and serum IL-6 in AIS patients

Increasing evidence suggested that inflammation plays critical roles in the development of cardiovascular diseases [32–34]. The pro-inflammatory cytokine IL-6 and hs-CRP was reported to reflect the degree of brain ischemic damage and stroke [35–41]. In this study, the levels of plasma hs-CRP and serum IL-6 were measured in AIS patients and control participants. The results showed that both plasma hs-CRP and serum IL-6 was significantly increased in the AIS patients in comparison with that of the control group (Fig. 5a and b). To further characterize the relationship between exosomal miR-134 and stroke, the correlation between the expressions of exosoma miR-134 and hs-CRP, IL-6 was analyzed, respectively. As shown in Fig. 5c and d, the level of miR-134 presented positive correlations with plasma hs-CRP and serum IL-6.

## Discussion

Aberrant expression of miRNAs is associated with the initiation and development of human diseases. Notably, the plasma exosomal miRNAs have been found as diagnosis biomarkers and potential therapeutic targets in some diseases, however, the expression of exosomal miRNAs in acute ischemic stroke and its correlation with the clinical characteristics of the stroke patients remain largely unknown. In this study, we isolated the exosomes from blood of AIS patients and found that exosomal miR-134 was highly expressed in the stroke patients compared with that of the control group. Additionally, miR-134 was positively correlated with the NIHSS score and poor prognosis of these patients. These findings suggested that potential involvement of exosomal miR-134 in AIS.

The intercellular communication is an essential process under both physiological and pathological conditions. It has been well documented that extracellular vesicles and exosomes are important platform to change the genetic information between cells [42]. The extraordinary value of exosomes in the diagnosis and treatment of stroke has drawn wide attention during recent years. The function of exosome in brain injury and neurondegenerative diseases has been demonstrated [43–48]. It has been reported that exosomes protected the brain tissues from ischemia-reperfusion injury after hypoxia precondition [49]. The protection role of exosomes secreted by the mesenchymal stem cells was achieved by delivering functional proteins or RNAs to neurons or astrocytes after ischemic injury [24, 50]. Previous study has showed that increased plasma microvesicles during cerebral ischemia [51, 52]. The number of microvesicles derived from endothelial cells was positively correlated with the occurrence of ischemic stroke [53, 54]. In the present study, increased exosomal miR-134 might indicate the enhanced production of exosomes in stroke patients, which responds to the intercellular brain injury. Further investigation is needed to explore the specific cell type or brain tissue where the exosome derived from.

MiRNAs have emerged as important regulators of cardiovascular physiological processes [55]. It has been documented that miR-21, miR-223 and miR-146b served as biomarkers for acute ischemic stroke [17, 56, 57]. Additionally, elevated serum expression of miR-15a, miR-16 and miR-17-5p was also found to be strongly associated with the AIS [58]. Recent findings showed that miR-134 regulated the ischemia injury-induced neuronal cell death [29]. In the present study, the exosomal miR-134 was significantly increased in AIS samples after stroke in comparison with that of the control group. No remarkable difference was observed for the level of exosomal miR-134 at the time point of 24-, 48- and 72 h after the stroke event. Further study is necessary to explore the alternation of exosomal miR-134 expression with relatively earlier time frame. In the present study, as blood samples of AIS patients were collected within 24 h after the stroke onset, we couldn’t rule out the possibility that the increase of exosomal miR-134 is due to the stroke event based on the present data. Therefore, more evidence is needed to confirm the conclusion that exosomal miR-134 is a potential independent risk factor in AIS. It might be helpful to detect the expression of exosomal miR-134 with normal subjects and follow the occurrence of AIS to establish the correlation between exosomal miR-134 and AIS. In principle, blocking the secretion and uptake of exosomes or inhibiting the expression of exosomal miR-134 might be necessary to investigate the involvement of exosomal miR-134 in the occurrence of AIS.

To evaluate the potential diagnostic value of exosomal miR-134 in AIS, receiver operating characteristic curve was analyzed and the data indicated that exosomal miR-134 might serve as a potential biomarker for distinguishing AIS from non-stroke control. Higher exosomal miR-134 expression may be an important indicator to be considered in medical examination, which might benefit the prevention and implement effective solutions to decrease the occurrence of AIS. To better provide the implication of this study, a larger sample size is necessary in the further investigation. Additionally, our data elucidated the positively correlation between the expression of exosomal miR-134 with the poor prognosis of AIS patients. Increasing evidence suggested that inflammation is involved in the progression of cerebrovascular diseases [59–62]. The plasma hs-CRP and serum IL-6 are important markers of inflammation and associated with the development of stroke. Consistently, our results found that serum exosomal miR-134 was significantly correlated with the increased expression of hs-CRP and IL-6 in AIS patients, which supported the conclusion that exosomal miR-134 was potential candidate for AIS prediction. As the function of miRNAs is achieved via modulating the expression of downstream target genes, further studies will be designed to explore the underlying molecular mechanism by which exosomal miR-134 in response to the ischemic stroke.

## Conclusions

Our study demonstrated that the expression of exosomal miR-134 in AIS patients was significantly increased. Enhanced abundance of the exosomal miR-134 was significantly correlated with the poor prognosis of AIS. Due to the relative small sample sizes in this study, further large-scale prospective data and longer follow-up studies were quite necessary to confirm the specificity and accuracy of exosomal miR-134 as a biomarker in the diagnosis of AIS.

## Abbreviations

AIS: Acute ischemic stroke
ANOVA: One-way analysis of variance
HRP: Horse radish peroxidase
hs-CRP: High-sensitivity C relative protein
IL-6: Interleukin 6
miRNAs: microRNAs
MRI: Magnetic resonance imaging
NIHSS: National Institutes of Health Stroke Scale
ROC: Receiver operating characteristic
RT: Room temperature
TEM: Transmission electron microscopy
TOAST: Trial of Org 10,172 in Acute Stroke Treatment
